# Clinical characteristics of a group of deaths with COVID-19 pneumonia in Wuhan, China: a retrospective case series

**DOI:** 10.1186/s12879-020-05423-7

**Published:** 2020-09-22

**Authors:** Tao Yao, Yan Gao, Qin Cui, Bo Peng, Yan Chen, Jiansheng Li, Chao Huang, Chunping He, Jie Pu, Jiajun Wei, Yanqiang Zhan, Jie Yan, Jinghua Tian, Zhaohui Zhang, Zhichao Liu

**Affiliations:** 1 Union Department of Infection Disease, Wuhan University Renmin Hospital, Wuhan, 430060 China; 2 Department of Neurology, Wuhan University Renmin Hospital, Wuhan, 430060 China; 3 Department of Gastroenterology, Wuhan University Renmin Hospital, Wuhan, 430060 China; 4 Department of Critical Care Medicine, Wuhan University Renmin Hospital, Wuhan, 430060 China; 5 Department of Obstetrics and Gynecology, Wuhan University Renmin Hospital, Wuhan, 430060 China

**Keywords:** Characteristics, COVID-19, Pneumonia, Death

## Abstract

**Background:**

With the widespread outbreak of novel coronavirus diseases 2019(COVID-19), more and more death cases were reported, however, limited data are available for the patients who died. We aimed to explore the clinical characteristics of deaths with COVID-19 pneumonia.

**Methods:**

We abstracted and analyzed epidemiological, demographic, clinical, and laboratory data from 83 death cases with COVID-19 pneumonia in East Hospital of Wuhan University Renmin Hospital, between January 26, 2020, and February 28, 2020.

**Results:**

Of the 83 deaths, none was the medical staff. The mean age was 71.8 years (SD 13.2; range, 34–97 years) and 53(63.9%) were male. The median from onset to admission was 10 days (IQR 7–14: range, 2–43 days), to death was 17 days (IQR 14–21: range, 6–54 days). Most deaths (66[80%]) had underlying comorbid diseases, the most of which was hypertension [47(57%)]. The main initial symptoms of these 83 deaths were shortness of breath(98.8%), fever(94%), and myalgia or fatigue(90.4%). Laboratory analyses showed the lymphocytopenia in 69(83%) deaths, hypoalbuminemia in 77(93%) deaths, the elevation of lactate dehydrogenase in 79(95%) deaths, procalcitonin in 69(83%) deaths and C-reactive protein in 79(95%) deaths. All 83 patients received antiviral treatment, 81(97.6%) deaths received antibiotic therapy, 54(65.1%) deaths received glucocorticoid therapy, and 20(24.1%) patients received invasive mechanical ventilation.

**Conclusion:**

Most of the deaths with COVID-19 pneumonia were elderly patients with underlying comorbid diseases, especially those over 70 years of age. The time of death after the onset of the disease was mostly 15–21 days. More care should be given to the elderly in further prevention and control strategies of COVID-19.

## Background

The novel coronavirus diseases 2019(COVID-19) first reported in Wuhan, Hubei province, China [1, 2]. It then spread widely in China and other nations around the world [3, 4]. With the COVID-19 global pandemic, more and more cases of death were reported [5–7]. As of July 1, 2020, 10,358,119 cases of COVID-19 have been confirmed and 508,085 of them died according to WHO Coronavirus Disease (COVID-19) Dashboard [8].

The previous studies have already reported the features of severe cases and death cases of COVID-19 [3, 9–11]. The result of a study from Wuhan in China showed that compared with survivors with COVID-19 pneumonia, non-survivors were older, more likely to develop ARDS, and more likely to receive mechanical ventilation, either invasively or non-invasively [9]. A survey from New York City in the United States found that older age, chronic cardiac disease, chronic pulmonary disease, higher concentrations of interleukin-6, and higher concentrations of D-dimer were independently associated with in-hospital mortality [10, 11]. As the number of patients confirmed COVID-19 and deaths continue to increase worldwide, reduce patient mortality, and improve prognosis has become an emergent issue confronting the current epidemic situation [8, 10, 11]. Further studies on the clinical features of death cases are needed, which could be valuable for the early specific management of critical patients.

In this study, we retrospectively collected and described detailed epidemiological, demographic, clinical, and laboratory characteristics of 83 deaths with COVID-19 pneumonia who had been admitted to East Hospital Wuhan University Renmin Hospital, which was one of the first designated hospitals in Wuhan to admit severe patients with COVID-19.

## Methods

### Study design and patients

This is a single-center retrospective study. We reviewed all patients with COVID-19 pneumonia who were admitted to East Hospital of Wuhan University Renmin Hospital as of February 28, 2020, and collected data on death cases in hospital. East Hospital of Wuhan University Renmin Hospital located in Wuhan, Hubei Province, China, was designated as one of the first hospitals to admit severe adult patients with COVID-19 pneumonia by government. The diagnostic standard of COVID-19 pneumonia is based on the 4th edition Protocol of Novel coronavirus pneumonia Prevention and Control Program issued by the National Health Commission of the People’s Republic of China [12].

This study protocol complied with the Medical Ethical Committee of Wuhan University Renmin Hospital (No.WDYR2020-k050). Written informed consent was waived due to the rapid emergence of this infectious disease.

### Data collection

Several investigators reviewed the electronic medical record system of the hospital, and abstracted epidemiological, demographic, clinical, and laboratory data from death cases with COVID-19 pneumonia as of February 28, 2020. The other two researchers reviewed and checked the data collected. The investigators directly contacted their families to refine the data if some epidemiological data of patients were not available in the medical record.

Nasopharyngeal swabs were obtained from all patients at admission. All samples were processed at the Department of Clinical Laboratory of Wuhan University Renmin Hospital. COVID-19 was confirmed by real-time polymerase chain reaction testing according to WHO guidelines for laboratory testing [13]. Positive confirmed patients with COVID-19 infection were defined as at least 2 positive test results and the detection interval should be at least 24 h.

### Statistical analysis

Continuous variables are expressed as the means ± standard deviations (SD) if they are normally distributed or medians (interquartile ranges, IQR) if they are not. Categorical variables are expressed as frequencies and percentages. All statistical analysis was performed with SPSS, version 25.0 (SPSS Inc., Chicago, IL, USA).

## Results

As of February 28, 2020, 83(5.7%) of the 1439 patients with COVID-19 pneumonia admitted to the hospital, died in hospital. None of the 83 deaths were medical staff, and there was no definite exposure history of patients with suspected or confirmed COVID-19.

The mean age was 71.8 years (SD 13.2; range, 34–97 years), including 26 patients over 80 years (31%) and 2 patient under 40 years (2%). Among the deaths, 53(63.9%) were male. The initial symptoms of these 83 patients were shortness of breath(98.8%), fever(94%), myalgia or fatigue(90.4%), anorexia(82%), cough(60.2%), hemoptysis(6%), pharyngalgia(6%), headache(3.6%), nausea or vomiting(2.4%) and diarrhea(4.8%) **(**Table 1**).**

**Table 1:** Demographics and clinical characteristics of 83 deaths with COVID-19 pneumonia

Characteristics	Patients, n(%)
***Demographic factors***	
Age(years) Mean (SD),	71.8(13.2)
< 40	2(2%)
40–49	3(4%)
50–59	10(12%)
60–69	15(18%)
70–79	27(33%)
≥ 80	26(31%)
Sex	
Men	53(63.9%)
Women	30(36.1%)
**Clinical Characteristics**	
Signs and symptoms at onset	
Fever	78(94%)
Myalgia or fatigue	75(90.4%)
Cough	50(60.2%)
Pharyngalgia	5(6.0%)
Headache	3(3.6%)
Haemoptysis	5(6.0%)
Shortness of breath	81(98.8%)
Anorexia	70(84.3%)
Nausea or Vomiting	2(2.4%)
Diarrhoea	4(4.8%)

Data are n (%) or mean (SD)

**Fig. 1:**
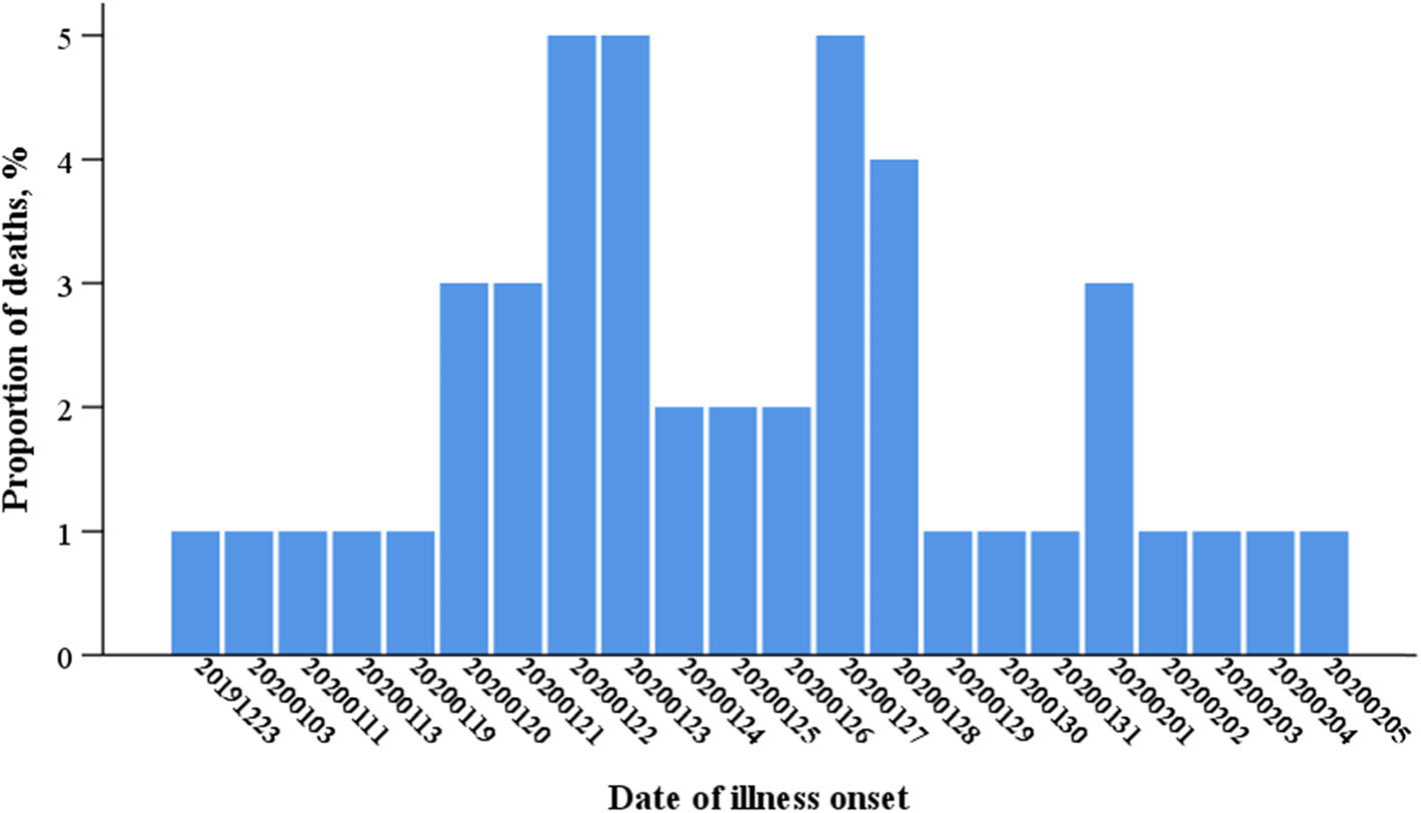
Date of illness onset of 83 deaths with COVID-19 pneumonia

Onset-to-admission interval of the 83 deaths was between 2 and 43 days (median 10 days, IQR 7–14), most of them were 6–10 days (43%). Onset-to-death interval was between 6 and 54 days (median 17 days, IQR 14–21), and most of them were 15–21 days for 50% of women and 45% of men **(**Table 2**)**. Figure 1 showed the date distribution of illness onset in all 83 patients. As described in this figure, the most dates of illness onset are between January 20 and January 28, 2020.

**Table 2:** Key Epidemiologic Variables of 83 deaths with COVID-19 pneumonia

Variable	Patients, n(%)
***Onset-to-admission interval***	10(7–14)
1–5 d	11(13%)
6-10d	36(43%)
10–15 d	25(30%)
≥16 d	11(13%)
***Onset-to-death interval***	17(14–21)
Women	16(14–20)
1-7d	1(3%)^a^
8–14 d	8(27%)^a^
15-21d	15(50%)^a^
22–28 d	4(13%)^a^
≥29 d	2(7%)^a^
Men	18(14–23)
1-7d	2(4%)^b^
8–14 d	13(25%)^b^
15-21d	24(45%)^b^
22–28 d	11(21%)^b^
≥29 d	3(6%)^b^

Data are median (IQR) or n (%). ^a^Proportion of women; ^b^Proportion of men

Of the 83 deaths, 66 patients (80%) had chronic comorbidities, the most of which was hypertension(57%), followed by cardiovascular disease(31%), diabetes (26%), cerebrovascular disease(17%), chronic lung disease(19.3%), chronic renal disease (6%), malignancy (6%) and chronic liver disease (4%) **(**Table 3**).**

**Table 3:** Characteristics of comorbidity of 83 deaths with COVID-19 pneumonia

	Patients, n(%)
Comorbidities	66(80**%**)
Hypertension	47(57**%**)
Diabetes	14(26**%**)
Cardiovascular	26(31**%**)
Cerebrovascular disease	14(17**%**)
Malignancy	5(6**%**)
Chronic lung disease	16(19**%**)
Chronic renal disease	5(6**%**)
Chronic liver disease	3(4**%**)
Number of comorbidities	
1	28(34**%**)
2	17(21**%**)
3	14(17**%**)
4	6(7**%**)
5	1(1**%**)

The main laboratory findings of the deaths on admission were shown in Table 4**.** The results of the blood count showed that white blood cell count in 5(6%) patients, lymphocyte count in 69(83%) patients, hemoglobin in 34(41%) patients, and platelets in 24(29%) patients were below the normal range. Also, white blood cell count in 34(41%) patients and mononuclear leucocyte in 16(19%) patients were above the normal range.

**Table 4:** Laboratory findings of 83 deaths with COVID-19 pneumonia on admission

Variables	Patients, n(%)
***Blood routine***	
White blood cell count (×10^9^/L; normal range 3.5–9.5)	9.1(4.7)
Decreased	5(6%)
Increased	34(41%)
Neutrophil count (×10^9^/L; normal range 1.8–6.3)	6.9(4.4–11.5)
Increased	49(59%)
Lymphocyte count (×10^9^/L; normal range 1.1–3.2)	0.6 (0.4–0.9)
Decreased	69(83%)
Mononuclear leucocyte(×10^9^/L; normal range 0.1–0.6)	0.4(0.3–0.5)
Increased	16(19%)
Hemoglobin (g/L; normal range 130–175 for men; 115–150 for women)	116 (104–121)
Decreased	34(41%)
Platelets (×10^9^/L; normal range 125–350)	166(72)
Decreased	24(29%)
***Blood biochemistry***	
Total bilirubin (μmol/L; normal range 0.0–23.0)	14.1(9.8–19.9)
Increased	16(19%)
Direct bilirubin (μmol/L; normal range 0.0–8.0)	5.6(4.2–9.3)
Increased	29(35%)
Aspartate aminotransferase (U/L; normal range 15.0–40.0)	43(28–62)
Increased	47(57%)
Alanine aminotransferase (U/L; normal range 7.0–40.0)	25(19–49)
Increased	25(30%)
Alkaline phosphatase (U/L; normal range 50–135)	76(59–105)
Increased	12(14%)
Gamma-glutamyl transpeptidas (U/L; normal range 7–45)	44(23–75)
Increased	41(49%)
Albumin (g/L; normal range 40–55)	33.7(4.1)
Decreased	77(93%)
Globulin (g/L; normal range 20–40)	25.6(22.6–29.0)
Decreased	4(5%)
Increased	2(2%)
Lactate dehydrogenase (U/L; normal range120–250)	493(362–682)
Increased	79(95%)
Serum creatinine (μmol/L; normal range 41–81)	77(55–113)
Increased	39(47%)
Blood urea nitrogen (mmol/L; normal range 3.1–8.8)	9.36(5.50–16.00)
Increased	44(53%)
***Coagulation function***	
Prothrombin time (s; normal range 9.0–13.0)	12.9(12.2–14.2)
Increased	36(43%)
Activated partial thromboplastin time (s; normal range 25.0–31.3)	29.1(27.1–32.5)
Increased	27(33%)
D-dimer (mg/L; normal range ≤ 0.55)	4.68(1.09–18.00)
Increased	78(94%)
***Inflammatory biomarkers***	
Procalcitonin (ng/mL; normal range ≤ 0.10)	0.23(0.12–0.94)
Increased	69(83%)
C-reactive protein (mg/mL; normal range ≤ 10.0)	85(47–180.0)
Increased	79(95%)
Interleukin-6 (pg/mL; normal range ≤ 20.0) ^**a**^	57.1(38.2–137.6)
Increased	19/23(82.6%)
ESR (mm/h; normal range ≤ 15) ^**b**^	39.75(32.10–63.40)
Increased	25/36(69.4%)
***Cardiac biomarkers***	
Hypersensitive cardiac troponin I (ng/mL; normal range ≤ 0.04) ^**c**^	0.27(0.09–1.07)
Increased	30/48(62.5%)
NT-pro B-type natriuretic peptide (pg/ml; normal range ≤ 900) ^**d**^	872.0(457.2–1914.5)
Increased	19/42(45.2%)
***Blood gas characteristics*** ^**e**^	
PH (normal range 7.35–7.45)	7.44(7.39–7.48)
PH < 7.35	13/57(22.8%)
PH > 7.45	24/57 (42.1%)
PaO_2_ (mmHg; normal range 80–100)	58.3(43.4–75.9)
PaO_2_ < 60 mmHg	44/57 (77.2%)
PaCO_2_ (mmHg; normal range 35–45)	34.1 (28.5–37.8)
PaO_2_ < 35 mmHg	33/57 (57.9%)
PaO_2_ > 50 mmHg	9/57 (15.8%)
Standard bicarbonate (mmol/L; normal range 21.0–25.0)	20.5 (17.8–24.4)

Data are mean (SD), median (IQR), and n (%). Decreased means over the upper limit of the normal range and increased means below the lower limit of the normal range

^a^ Data available for 23 of 83 patients; ^b^ Data available for 36 of 83 patients; ^c^ Data available for 48 of 83 patients; ^d^ Data available for 42 of 83 patients; ^e^ Data available for 57 of 83 patients

On admission, many patients had an abnormal liver function and renal function. Aspartate aminotransferase in 57% patients, gamma-glutamyl transpeptidase in 49% patients, serum creatinine in 47% patients, and blood urea nitrogen in 53% patients were above the normal range. Albumin levels were lower than normal in 93% patients. Lactate dehydrogenase increased in 95% patients. Most patients have abnormal coagulation function, which showed the elevation of D-dimer in 94% patients, the extension of Prothrombin time in 43% patients, and Activated partial thromboplastin time in 33% patients. Moreover, procalcitonin (83%) and C-reactive protein (95%) increased above the normal range in most patients.

**Fig. 2:**
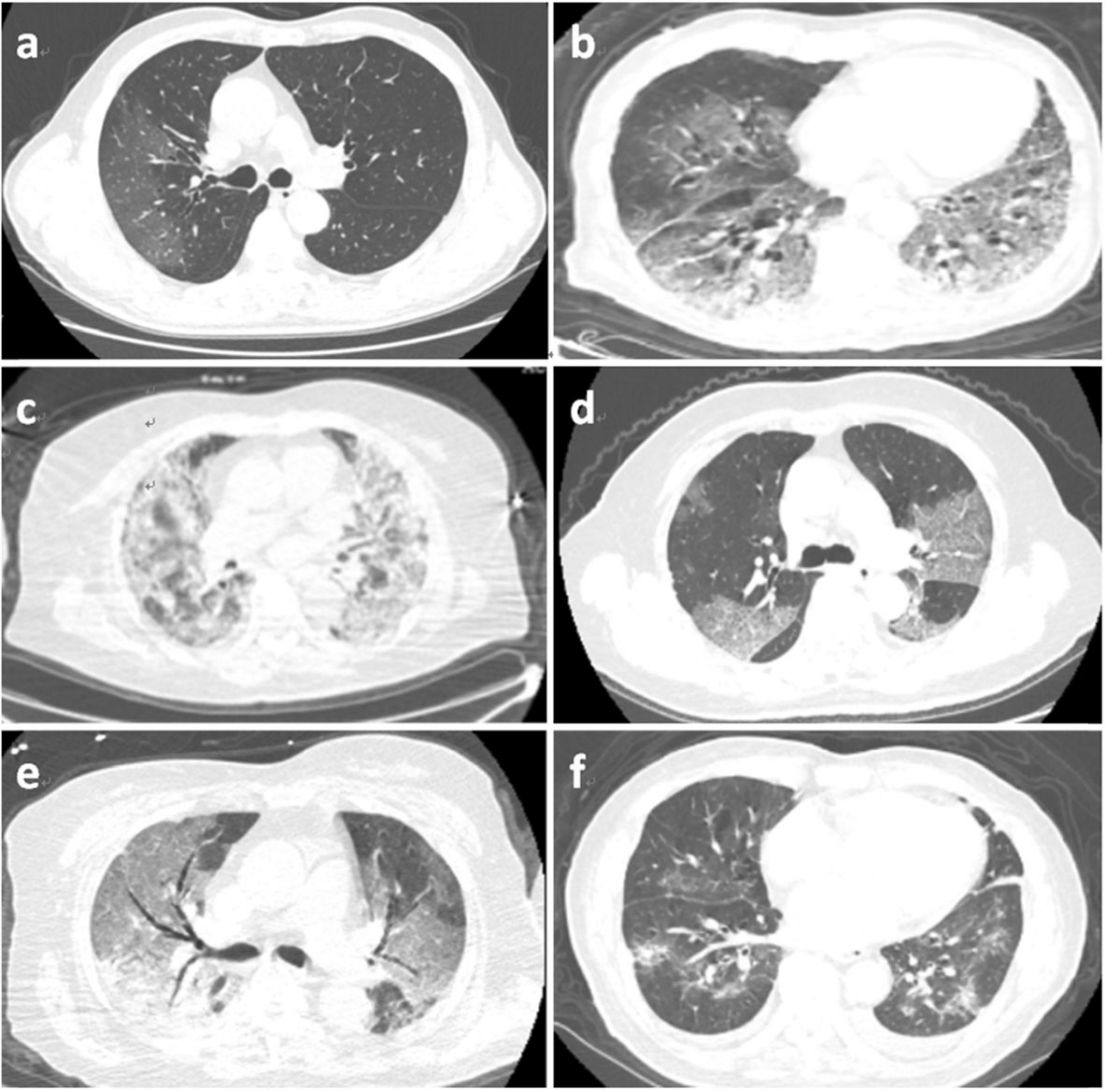
Lung imaging features of axial chest CT scans. **a** Ground glass opacity(GGO) on the right lung in a patient between 60 and 69 years of age; **b** Multiple GGO and bronchial dilatation of both lungs in a patient between 60 and 69 years of age; **c** Multiple GGO and consolidation in a patient between 60 and 69 years of age; **d** Multiple GGO of both lungs and local consolidation in a patient between 70 and 79 years of age; **e** Multiple GGO and air bronchogram in a patient between 60 and 69 years of age; **f** Multiple GGO and pleural effusion in a patient between 80 and 89 years of age

Each patient performed a chest CT scan on admission, and pneumonia was confirmed in all 83 patients, and 71 patients were involved in the bilateral lung (Table 5). Radiographic features from chest CT scans mainly included ground glass opacity, consolidation, air bronchogram, bronchial dilatation, and pleural effusion or thickening. Multiple patchy ground-glass shadows were the main feature in the chest CT of most patients, followed by consolidation (Table 5**;** Fig. 2).

**Table 5:** Main lung imaging features on chest CT images of 83 deaths with COVID-19 pneumonia on admission

	Patients, n(%)
***Lung imaging features***	
Unilateral pneumonia	12(14.5%)
Bilateral pneumonia	71(85.5%)
Ground-glass opacity	83(100%)
Consolidation	33(39.8%)
Air bronchogram	18(21.7%)
Bronchial dilatation	23(27.7%)
Pleural effusion or thickening	47(56.6%)

The main complications were acute respiratory failure(85.5%), sepsis (83.1%), heart failure(44.6%), acute kidney injury (26.5%), gastrointestinal bleeding(19.2%), acute liver injury(10.8%), acute myocardial infarction(7.2%) (Table 6) In drug treatment, all 83 patients received monotherapy and combination antiviral therapy for 5–10 days, and all of them received Abidole, 58 received Oseltamivir, 16 received Ribavirin, 4 received Lopinavir and Ritonavir. Among them, 57 (68.7%)patients received Abidole and Oseltamivir, 16(19.3%) received Abidole and Ribavirin, 3(3.6%) received Abidole and Lopinavir and Ritonavir, and 1(1.2%) received Lopinavir, Oseltamivir and Lopinavir and Ritonavir. Of 83 patients, 81(97.6%) patients received antibiotics therapy including Penicillins(28.9%), Cephalosporins(62.7%), Quinolone(42.4%) and Vancomycin(13.3%). 54(65.1%) patients received glucocorticoid therapy and 39(47.0%) patients received intravenous immunoglobulin therapy. In addition to drug treatment, all patients received oxygen therapy, 20 of them(24.1%) received invasive mechanical ventilation(IMV), 51 of them(61.4%) received non-invasive mechanical ventilation(NIMV), and 12 of them(14.5%) received high flow nasal cannula. 6(7.3%) patients received continuous replacement therapy(CRRT) due to severe renal dysfunction, 1(1.2%) patients were given extracorporeal membrane oxygenation treatment(ECMO) (Table 6).

**Table 6:** Bacterial culture results, complications, and treatment of 83 deaths with COVID-19 pneumonia after admission

	Patients, n(%)
***Bacterial culture results***	36(43.4%)
Sputum bacteria culture positive	22(26.5%)
Blood bacteria culture positive	9(10.8%)
Urine bacteria culture positive	5(6.2%)
Fecal bacteria culture positive	0
***Complications***	
Acute respiratory failure	71(85.5%)
Acute myocardial infarction	6(7.2%)
Heart failure	37(44.6%)
Acute liver injury	9(10.8%)
Acute kidney injury	22(26.5%)
Gastrointestinal bleeding	16(19.2%)
Sepsis	69(83.1%)
Shock	33(39.8%)
***Treatments***	
Antiviral therapy	83(100%)
Monotherapy	
Abidole	6(7.2%)
Combination therapyAbidole	
Abidole/ Oseltamivir	57(68.7%)
Abidole/ Ribavirin	16(19.3%)
Abidole/ Lopinavir and Ritonavir	3(3.6%)
Abidole/ Oseltamivir/ Lopinavir and Ritonavir	1(1.2%%)
Antifungal therapy	2(2.4%%)
Antibiotic therapy	81(97.6%)
Penicillins	24(28.9%)
Cephalosporins	52(62.7%)
Quinolone	35(42.2%)
Vancomycin	11(13.3%)
Glucocorticoid therapy	54(65.1%)
Intravenous immunoglobulin therapy	39(47.0%)
CRRT	6(7.3%)
Invasive mechanical ventilation	20(24.1%)
Non-invasive mechanical ventilation	51(61.4%)
High flow nasal cannula	12(14.5%)
ECMO	1(1.2%)

*CRRT* continuous renal replacement therapy, *ECMO* extracorporeal membrane oxygenation

## Discussion

This retrospective study described the epidemiological and clinical characteristics of 83 deaths with COVID-19 pneumonia. To my knowledge, our study is the first epidemiological investigation, whose subjects were all patients with COVID-19 pneumonia who died.

In this study, the median of the onset-to-admission interval was longer than that of patients in the previous two studies [9, 14]. Most patients were hospitalized more than 6 days after the onset of the disease, and the longest was 43 days. Two factors likely contributed to the interval. Fisrt, some patients have no severe symptoms in the early stage and it took more for home isolation and community treatment. Second, due to the COVID-19 outbreak, the isolation ward of the hospital may have been under capacity in the initial. Most of the patients who died in the 15-21 days after the onset of the disease, both male and female. This result indicates that the third week may be a period of a high risk of death for critically ill patients with COVID-19.

Most of the deaths with COVID-19 pneumonia were elderly patients especially those over 70 years of age, and male patients. These are consistent with a recent study [9]. The cases of COVID-19 in pregnant women have been mentioned in the previous study [15]. Of 29 pregnant women with COVID-19 in the hospital, there are no deaths so far.

The proportion of patients with comorbidities was higher than previous studies in patients with COVID-19 [9, 14, 16]. The most common comorbidities were hypertension in our study, which was diabetes in two previous cohort studies of Middle Eastern respiratory syndrome coronavirus (MRSE-Cov) infection, and severe acute respiratory syndrome coronavirus(SARS-CoV) infection [17, 18]. We observed that the majority of patients who died were also geriatric patients and those suffering from chronic comorbidities. However, some healthy people(22%) died without complications, which were an indication of the high pathogenicity of COVID-19. The initial clinical symptoms of patients infected with COVID-19 were nonspecific. There were no significant differences in the types of initial symptoms between the deaths in our study and the recently published studies [9, 14, 16, 19]. However, the first three symptoms in our study were shortness of breath, fever, myalgia, or fatigue. A small number of patients initially presented with gastrointestinal symptoms, such as anorexia, nausea, vomiting, and diarrhea, which were mentioned in previous studies [9, 16].

Most of the patients in our study developed acute respiratory failure(ARF) including ARDS, fatal infection, abnormal coagulation, and eventually multiple organ failure, except for six who died of acute myocardial infarction. 12 (14.5%) patients with unilateral pneumonia at admission were dead in our study. We analyzed the causes of death of these patients are as follows: First, the patient could progress to bilateral lung infection and even severe ARF after admission. Secondly, some patients have severe comorbidities such as chronic heart failure, chronic lung disease, chronic renal disease. Finally, some patients develop serious complications such as acute myocardial infarction, multiple organ failure.

As a newly identified disease, little is known about the pathogenic mechanism of COVID-19. Most of the patients who died had abnormal coagulation. Increased inflammatory markers such as procalcitonin and C-reactive, lymphopenia were a common characteristic in the patients. This series of changes is a manifestation of the immune response and maybe be a factor in poor prognosis [9, 14, 18]. In a recent fatal case report, typical features of inflammation were observed in the pulmonary pathology of the patient, whose pathological section showed interstitial mononuclear inflammatory infiltrates, dominated by lymphocytes [20]. These pathological characteristics greatly resemble those of MRSE-Cov infection and SARS-CoV infection [20–22].

Until now, no drugs are specifically effective against coronaviruses. Of the 83 patients in our study, each patient received Abidol, and some patients were treated with Oseltamivir, Ganciclovir, Lopinavir, and Ritonavir, but none of them had a definite therapeutic effect. Besides, Radcivir is an unlisted nucleotide drug whose broad-spectrum antiviral activity has been confirmed in animal models [23, 24]. It may be a potentially effective drug for patients with COVID-19 [20]. Two randomized controlled clinical trials (NCT04252664; NCT04257656) to assess the safety and efficacy of Radcivir are is currently underway in patients hospitalized with COVID-19 pneumonia.

The patients in this study were generally had associated with a secondary bacterial infection, followed by sepsis and septic shock. 97.6% of the patients were treated with antibiotics based on abnormal inflammatory markers and bacterial culture results.

In patients with SARS and MERS, the effect of glucocorticoid therapy on prognosis is controversial [25, 26]. However, severe patients with COVID-19 may be beneficial from glucocorticoid therapy to prevent ARDS development, based on recent studies [16, 20]. 65.1% of the patients in this study received glucocorticoid therapy.

Critical illness among patients hospitalized with COVID-19 is common and associated with a high frequency of IMV in some recent large sample studies [10, 27, 28]. In our study, 63 patients did not receive IMV, 41 of whom declined IMV. Of the 41 patients who declined IMV, 9 patients declined IMV themselves, and 32 patients were completely incapacitated due to serious illness and the family members declined it to relieve the suffering of the patient considering the patient’s advanced age and poor prognosis. The other 22 patients did not receive IMV for unknown reasons lacking medical records, which did not rule out a medical run in the early stages of the COVID-19 outbreak. The timing of intubation in patients with severe COVID-19 pneumonia is challenging. Most patients with acute respiratory distress syndrome (ARDS) due to COVID-19 will warrant intubation and mechanical ventilation [9, 10, 28]. Delaying IMV until the patient acutely decompensates is potentially harmful to the patient and affects prognosis [29].

Our study has several limitations. First of all, the study had a limited number of cases, with only 83 deaths. However, to our knowledge, very few case series of deaths have been reported, the data is a valuable demonstration of characteristics of deaths with COVID-19 pneumonia in the early period of exponential growth. Secondly, some data such as cytokines (eg, IL2, IL4, IL6, IL10, TNF, IFN γ) were absent in patients admitted early, which were related to lung injury in previous studies SARS-CoV and MERS-CoV [30, 31]. We will routinely observe the changes of cytokines of patients in further study. Thirdly, the study has not compared the difference between surviving and dead patients due to the initial design. However, this is a series of study designs and the patients will continue to be followed up.

## Conclusions

This single-center retrospective case series early shows the epidemiological and clinical characteristics of deaths with COVID-19 pneumonia. Most of the deaths with COVID-19 pneumonia were elderly patients with underlying comorbid diseases, especially those over 70 years of age. The time of death was mostly 15–21 days after the onset of the disease. More care should be given to the elderly in further prevention and control strategies of COVID-19.

## Data Availability

The data will be available from the corresponding author on a reasonable request. After the publication of this study, the participant data without names an identifiers will be made available after approval from the corresponding authors and Wuhan University Renmin Hospital.

## Abbreviations

COVID-19: Novel coronavirus diseases 2019()
SD: Standard deviations
IQR: Interquartile ranges
GGO: Ground glass opacity
CRRT: Continuous renal replacement therapy
IMV: Invasive mechanical ventilation
ECMO: Extracorporeal membrane oxygenation
MRSE-Cov: Middle Eastern respiratory syndrome coronavirus
SARS-CoV: Severe acute respiratory syndrome coronavirus
ARF: Acute respiratory failure
WHO: World health organization
